# When cognitive offloading becomes dependence: how AI dependence mediates the pathway from academic stress to burnout and anxiety

**DOI:** 10.1186/s40359-026-04911-w

**Published:** 2026-05-30

**Authors:** Wenlong Wang, Yuhang Wu, Jie Fang, Chong Yang, Langyi Wen

**Affiliations:** 1 Psychological Counselling Center, Guangdong University of Finance and Economics, Guangzhou, 510320 China; 2 Institute of Development and Reform, Guangdong University of Finance and Economics, Guangzhou, 510320 China; 3 School of Big Data and Artificial Intelligence, Guangdong University of Finance and Economics, Guangzhou, 510320 China

**Keywords:** AI dependence, Academic stress, Self-efficacy, Academic burnout, Anxiety, Cognitive offloading, College students

## Abstract

Although generative AI tools are increasingly used in college students’ academic activities, their role in stress-related maladaptive coping remains unclear. This study focuses specifically on maladaptive AI dependence as a potential mediator linking academic stress to academic burnout and anxiety. A total of 1,623 college students completed an online survey using the Academic Stress Scale, the AI Dependence Scale, the General Self-Efficacy Scale, the Academic Burnout Scale, and the GAD-7. PROCESS Model 6 was used to examine direct, indirect, and sequential mediation effects separately for academic burnout and anxiety. The results showed that academic stress positively predicted both academic burnout and anxiety. Sequential mediation analyses revealed that academic stress was associated with increased AI dependence, which may reduce students’ mastery experiences and encourage reliance on external support, thereby undermining self-efficacy and ultimately contributing to higher levels of burnout and anxiety. These findings suggest that excessive reliance on AI as a stress-coping strategy may weaken students’ sense of competence and increase their vulnerability to academic burnout and anxiety. Educational interventions should therefore promote reflective and autonomy-supportive AI use, positioning AI as a learning scaffold rather than a substitute for students’ own cognitive engagement.

## Introduction

In recent years, the learning style of college students has been dramatically changed with the widespread use of Generative Artificial Intelligence (GAI), represented by ChatGPT, Gemini, and DeepSeek. These artificial intelligence applications are able to write coherent texts, solve complicated problems, and give immediate responses; college students have widely used them in their routine academic activities such as searching for information, accomplishing assignments and preparing for exams. Although AI tools have since become a recognized efficient option to assist college students in coping with their academic tasks, there are growing anxieties about the psychological and cognitive effects of the excessive or maladaptive use of AI tools, in the context of increased academic stress [20, 46].

It is important to distinguish between AI use and AI dependence, which represent conceptually different constructs. AI use refers to the frequency or manner in which individuals engage with AI tools for academic tasks. In contrast, AI dependence reflects a qualitatively different level of engagement, characterized by reduced autonomy and increased reliance on AI for cognitive processing and problem-solving [1]. While AI use can be adaptive and supportive under certain conditions, AI dependence may function as a maladaptive coping strategy, particularly when individuals rely on AI as a substitute for their own cognitive efforts.

Cognitive offloading theory suggests that people increasingly rely on external tools to store, process, or generate information, thereby reducing the need for active cognitive engagement [30]. In the context of AI-assisted learning, excessive reliance on AI tools may lead students to substitute their own cognitive efforts with algorithmic outputs, potentially reshaping how they engage with academic tasks [11].

Although AI dependence is receiving increasing discussion in the context of education, empirical studies of psychological outcomes concerning AI dependence have been insufficient. Current research has focused on academic integrity, learning performance, or attitudes towards AI, as opposed to its role as a stress coping mechanism with mental health implications [9]. Furthermore, little attention has been paid to how academic stress can be an antecedent to AI dependence or how AI dependence can influence motivational variables such as self-efficacy to regulate negative academic outcomes, which include burnout and anxiety.

### Academic stress and its relations to academic burnout and anxiety

Academic stress is accumulated over a long period, including heavy coursework, frequent testing, competition, time pressure, and worry about future career opportunities [5, 27]. According to the transactional model of stress, stress is experienced when individuals perceive environmental demands exceed their available coping resources [16]. In academic practice, this appraisal process is important in determining emotional and behavioral responses of students, making academic stress becomes a major antecedent of maladaptive outcomes, including academic burnout and anxiety.

Academic burnout is typically viewed as a condition of emotional exhaustion, cynicism, or a lack of involvement with academic practice as well as a diminished perception of academic achievement [21, 34]. Empirical research consistently demonstrates that higher levels of perceived academic stress are associated with greater academic burnout among university students across diverse cultural contexts [19, 31]. Salmela-Aro and Upadyaya, [32] discovered that chronic stress caused by academic study predicted higher levels of exhaustion and disengagement over time. Notably, burnout has been attributed to various unfavorable academic phenomena, such as decreased motivation, academic performance, increasing absenteeism, and dropout intentions [22]. These results underscore the problem of academic burnout as one of the key indicators of damaged student well-being caused by incessant academic strain.

Anxiety is another psychological outcome of academic stress. According to meta-analytic studies, academic stress can be a strong predictor of anxiety symptoms in college students, and even surpass the effect of other non-academic life stressors [27]. Competition in tests, fear of failing, and unpredictability about academic performance are the main factors that lead to chronic anxiety, which can inhibit the ability to focus on learning material, working memory, and self-regulated learning [26]. Anxiety and burnout are not exclusive, they tend to co-exist and reinforce each other. Emotional exhaustion may increase anxiety by diminishing individual’s coping capacity, while chronic anxiety may accelerate burnout due to increasing emotional strain [7].

### The mediating roles of AI dependence and self-efficacy

According to the Cognitive Offloading Theory, individuals externalize cognitive processes to tools or technologies in order to reduce mental effort and manage limited cognitive resources [30]. College students under academic stress particularly tend to offload cognitive demands to AI tools that provide quick, organized, and seemingly authoritative answers. Recent research indicates that students are increasingly applying generative AI not only to request information, write, and solve problems, but also to outsource fundamental learning processes to AI systems [15, 44].

Related studies on technologies such as smartphones and search engines have indicated that frequent cognitive offloading is linked to less critical thinking, worse memory retrieval, and excessive reliance on external assistance [3, 38]. Therefore, AI dependence can be a maladaptive mediator between academic stress and negative academic and psychological consequences, such as burnout and anxiety.

One of the key psychological processes by which AI dependence can exert its effects is self-efficacy, which refers to the perception of one’s capacity to accomplish tasks and conquer challenges [2], and serves as the major predictor of motivation, perseverance, and emotional health in academic context. Previous research has revealed that higher academic self-efficacy is associated with better learning strategies, lower stress, reduced anxiety, and may protect students against academic burnout [14, 34].

Recent research suggest that engagement with generative AI may initially enhance perceived competence while simultaneously increasing reliance on external cognitive supports [43]. This dynamic creates a paradoxical effect where immediate task success masks a gradual decline in the development of autonomous skills.

This concern is supported by emerging empirical evidence. Several studies have indicated that frequent dependence on AI tools is related to lower perceived learning and less confidence in problem-solving skills, despite improvements in short-term performance [10, 15]. As students habitually offload core cognitive functions, they may experience fewer mastery experiences [45], thereby leading to a diminished self-efficacy.

From a broader perspective, the relationships between academic stress and downstream psychological outcomes are increasingly understood as being shaped through cognitively mediated processes. Recent research in higher education has demonstrated that academic outcomes are frequently structured through such indirect, anxiety-mediated pathways [42]. Accordingly, AI dependence and self-efficacy can be framed as critical elements embedded within this broader stress-processing mechanism.

### The present study

Despite growing research on generative AI in education, several important gaps remain. Most studies have focused on performance, integrity, or attitudes toward AI, with little attention to AI dependence as a psychological coping mechanism under academic stress. Moreover, the mechanisms linking academic stress to burnout and anxiety in AI-supported contexts remain unclear, particularly how AI dependence interacts with motivational factors like self-efficacy.

To address these gaps, the present study proposes an integrative framework grounded in the Cognitive Offloading Theory [30] and transactional model of stress and coping [16], exploring how academic stress may translate into academic burnout and anxiety in the contemporary AI-rich learning environment.

Based on existing empirical evidence and theoretical reasoning, the following hypotheses are proposed:

- Hypothesis 1 (H1). Academic stress will be positively related to academic burnout and anxiety.
- Hypothesis 2 (H2). AI dependence mediates the relationship between academic stress and academic burnout/anxiety.
- Hypothesis 3 (H3). Self-efficacy mediates the relationship between academic stress and academic burnout/anxiety.
- Hypothesis 4 (H4). AI dependence and self-efficacy play as chain mediators in the relationship between academic stress and academic burnout/anxiety.

## Method

### Participants and procedure

Participants were undergraduate students recruited from universities in China through an online survey platform. A convenience voluntary online sampling approach was adopted, and the questionnaire was distributed through social media platforms and university networks. All participants completed the questionnaire voluntarily and anonymously after providing informed consent.

Responses were excluded if participants failed the fixed-answer items (questions with obvious answers to detect casual respondents) or spent less than 2 min or more than 30 min on the entire questionnaire, indicating inattentive or invalid responding.

After the process, the total number of valid samples was 1623. To further assess the adequacy of the sample size, a post-hoc power analysis was conducted using G*Power 3.1.9.7. Assuming a small-to-medium effect size (*f* ² = 0.05), an alpha level of 0.05, and multiple predictors, the results indicated that the sample size of 1,623 provided statistical power greater than 0.99. This suggests that the present study had sufficient power to detect the hypothesized effects. Among the valid samples, 46.27% were male (751), and 53.73% were female (872). The average age of the sample was 19.47 years (SD = 1.08). In terms of academic characteristics, participants were drawn from different years of study and a range of academic disciplines, including social sciences, natural sciences, and engineering. This diversity enhances the representativeness of the sample within the higher education context.

### Measures

#### Academic stress

This study used the subscale of academic stress from the Stress Scale for College Students [17]. It includes 10 items and uses a 4-point Likert scale (1 = no stress, 4 = severe stress), with higher scores indicating higher academic stress. This scale has demonstrated with good reliability and validity and has been widely applied in relevant studies. In this study, the Cronbach’s α coefficient of this scale was 0.91.

#### AI dependence

The DAI scale, developed by [24], was employed to measure individuals’ dependence on artificial intelligence. The scale assesses AI dependence rather than general AI use, focusing on individuals’ reliance on AI for cognitive tasks and decision-making, rather than the frequency of tool usage. It incorporated advised criteria of compulsive behaviors or dependencies in DSM-5 [12]. This scale includes 5 items, such as “I feel unprotected when I do not have access to AI”, “I’m concerned about the idea of being left behind in my tasks or projects if I do not use AI”. The DAI scale was presented in a 5-point Likert format with response options ranging from 1 (completely false for me) to 5 (describes me perfectly). In this study, the Cronbach’s α coefficient of this scale was 0.76.

#### Self-efficacy

The General Self-Efficacy Scale (GSES) was used to measure the college students’ belief in their competence to cope with stressful tasks in study and campus life (Schwarzer & Jerusalem, [36]). This scale incorporates 10 items with a 4-point Likert format (1 = totally incorrect, 4 = totally correct), with a higher score indicating better self-efficacy. In this study the Cronbach’s α coefficient was 0.93.

#### Academic burnout

This study employed the Academic Burnout Scale developed by Lian et al., [18]. The scale measures three dimensions of academic burnout: low mood, misconduct, and low sense of achievement. It has 20 items with a 5-point Likert format (1 = completely disagree, 5 = completely agree), with a higher score indicating more significant academic burnout. In this study the Cronbach’s α coefficient of this scale was 0.95.

#### Anxiety

The scale of Generalized Anxiety Disorder (GAD-7) [39] was used, which consists of 7 items. A 4-point Likert format was applied (1 = not at all, 4 = almost every day), with higher scores indicating more severe anxiety symptoms. This scale has excellent reliability (Cronbach’s α = 0.92), and in this study, the Cronbach’s α coefficient for this scale was also 0.92.

### Statistical methods

Data were analyzed using SPSS 27.0. Prior to the main analyses, data screening procedures were conducted. Outliers were examined using standardized z-scores, with values exceeding ± 3 considered potential outliers. No extreme outliers were identified that required removal. In addition, cases with missing or invalid responses had already been excluded during the data cleaning stage.

Descriptive statistics and Pearson correlation analyses were conducted to examine the relationships among the key variables. To assess common method bias, Harman’s single-factor test was performed.

Before conducting inferential analyses, the assumptions of normality were evaluated. All variables fell within the acceptable range (skewness within ± 2, kurtosis within ± 7) [40], indicating no serious deviation from normality.

Multicollinearity was assessed using variance inflation factors (VIF), with all values below the recommended threshold of 5 (ranging from 1.08 to 1.19), and tolerance values were all above 0.10 (ranging from 0.84 to 0.93), indicating that multicollinearity was not a concern in this study.

To test the hypothesized mediation model, the Model 6 of PROCESS V4.2 macro plugin [13] was used for serial mediation analysis. This model allows for the examination of both independent and sequential mediating effects. In the present study, AI dependence was specified as the first mediator (*M*_*1*_) and self-efficacy as the second mediator (*M*_*2*_). Accordingly, the path from AI dependence to self-efficacy represents the *d*_*21*_ path in the serial mediation model. To enhance the precision of our estimates, gender, grade level, and academic major were treated as control variables in the mediation analysis.

Indirect effects were tested using a bootstrapping procedure with 5,000 resamples. Statistical significance was determined based on the 95% bias-corrected confidence intervals (LLCI and ULCI). An indirect effect was considered significant if the confidence interval did not include zero.

Two separate mediation models were estimated: one with academic burnout as the dependent variable and the other with anxiety as the dependent variable.

## Results

### Common method bias control

Before conducting the questionnaire, the order of instructions and items was controlled to reduce response resistance and bias caused by homogeneous measurement scales. As all the measurements in this study were self-report questionnaires, which may cause common method bias, Harman’s single-factor test was conducted. The results showed that the first factor explained 32.83% of the total variance, which is lower than the recommended threshold for a single dominant factor [41]. This indicates that there was no significant common method bias in this study.

### Descriptive statistics and correlations among variables

The means, standard deviations, and correlations among the key variables are presented in Table 1.

As shown in Table 1, academic stress was positively correlated with AI dependence (*r* = 0.26, *p* < 0.001), academic burnout (*r* = 0.59, *p* < 0 0.001) and anxiety (*r* = 0.56, *p* < 0 0.001), and negatively correlated with self-efficacy (*r* = -0.34, *p* < 0.001). AI dependence was positively correlated with academic burnout (*r* = 0.34, *p* < 0.001) and anxiety (*r* = 0.32, *p* < 0 0.001), and negatively correlated with self-efficacy (*r* = -0.16, *p* < 0 0.001). Self-efficacy was negatively correlated with academic burnout (*r* = -0.44, *p* < 0 0.001) and anxiety (*r* = -0.29, *p* < 0 0.001).

**Table 1:** Descriptive statistics and correlation matrix

Variable	Mean	SD	1	2	3	4	5
1. Academic stress	2.16	0.56	-	0.26***	-0.34***	0.59***	0.56***
2. AI dependence	2.98	0.74	-	-	-0.16***	0.34***	0.32***
3. Self-efficacy	2.48	0.61	-	-	-	-0.44***	-0.29***
4. Academic burnout	2.58	0.75	-	-	-	-	0.53***
5. Anxiety	1.60	0.58	-	-	-	-	-

* *p* < 0.05, ** *p* < 0.01, *** *p* < 0.001

### Mediation analysis of model 1

**Fig. 1:**
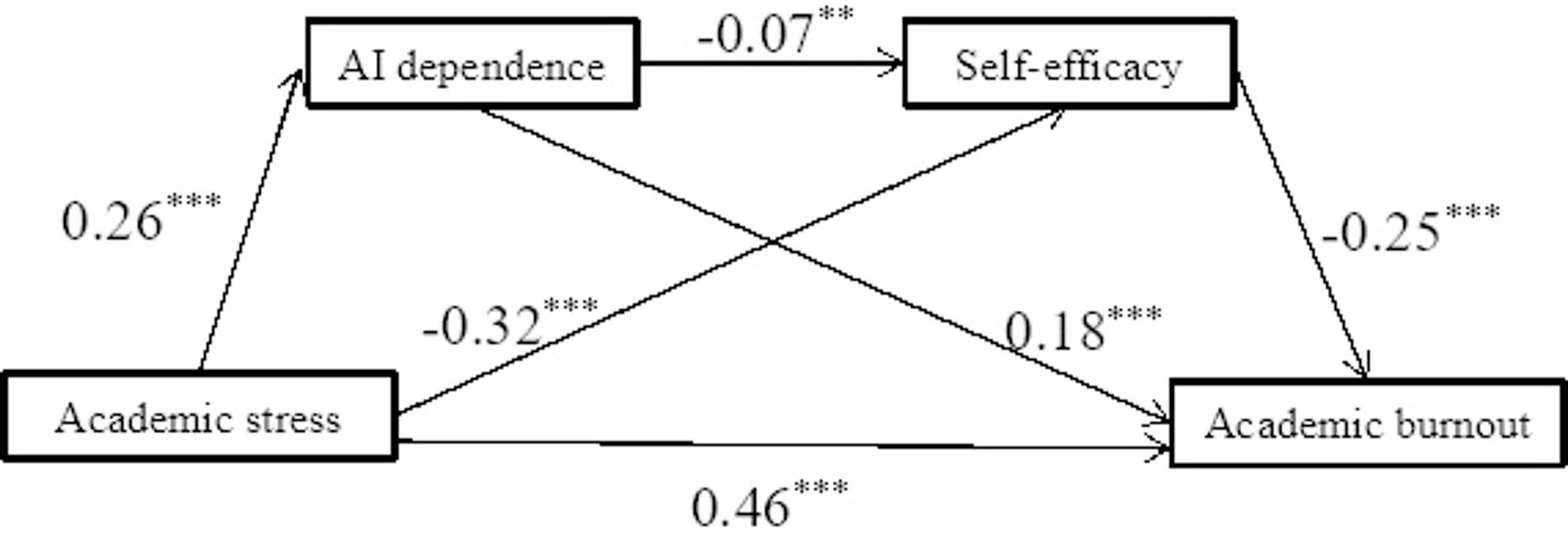
Paths of model 1 (the coefficients are standardized)

Model 1 examined the mediating roles of AI dependence and self-efficacy in the relationship between academic stress and academic burnout. The results of the path coefficients are shown in Fig. 1 and Table 2, and the mediation effects are presented in Table 3.

According to PROCESS V4.2, the 95% confidence interval for the mediating effect was estimated by extracting 5,000 bootstrap samples. The results showed that the regression model was significant, with R^2^ = 0.35, *F* (1, 1621) = 872.34, *p* < 0.001. Academic stress positively predicted AI dependence (*β* = 0.26, *p* < 0.001). AI dependence, in turn, negatively predicted self-efficacy (*β* = -0.07, *p* < 0.01). Finally, self-efficacy negatively predicted academic burnout (*β* = -0.26, *p* < 0.001).

**Table 2:** Results of the chain mediation model 1

Outcome	Predictor	*R*²	F	SE	t	β
Academic burnout	Academic stress	0.35	872.34***	0.267	29.535	0.592***
AI dependence	Academic stress	0.07	121.26***	0.031	11.01	0.264***
Self-efficacy	Academic stress	0.12	112.49***	0.026	-13.37	-0.323***
	AI dependence			0.020	-3.03	-0.073**
Academic burnout	Academic stress	0.44	431.88***	0.027	22.54	0.456***
	AI dependence			0.020	9.48	0.183***
	Self-efficacy			0.024	-12.89	-0.255***

*β *Standardized coefficient

* *p* < 0.05, ** *p* < 0.01, *** *p* < 0.001

**Table 3:** Mediation effect analysis of model 1

Effect Type	Path	Effect	BootSE	LLCI	ULCI
Direct Effect	Academic stress→Academic burnout	0.608	0.035	0.540	0.677
Indirect Effect	Academic stress→AI dependence→Academic burnout	0.064	0.010	0.045	0.086
	Academic stress→Self-efficacy→Academic burnout	0.110	0.015	0.081	0.141
	Academic stress→AI dependence→Self-efficacy→Academic burnout	0.007	0.003	0.002	0.012
Total Indirect Effect		0.180	0.018	0.146	0.217
Total Effect		0.788	0.031	0.727	0.848

Bootstrap results based on 5000 samples. CI = 95% Confidence Interval. If 0 is not between LLCI and ULCI, the effect is significant

#### Direct effect

The results indicated that the direct effect of academic stress on academic burnout was 0.61 (95% CI = [0.54, 0.68]), which remained significant after controlling for the mediators.

#### Indirect effects

The total indirect effect through AI dependence and self-efficacy was 0.18 (95% CI = [0.15, 0.22]). Specifically, this total indirect effect consists of three significant specific indirect pathways: (a) the indirect effect via AI dependence alone was 0.06 (95% CI = [0.05, 0.09]); (b) The indirect effect via self-efficacy alone was 0.11 (95% CI = [0.08, 0.14]); (c) the sequential indirect effect (academic stress→AI dependence→self-efficacy→academic burnout) was 0.01(95% CI = [0.002, 0.012]).

### Mediation analysis of model 2

**Fig. 2:**
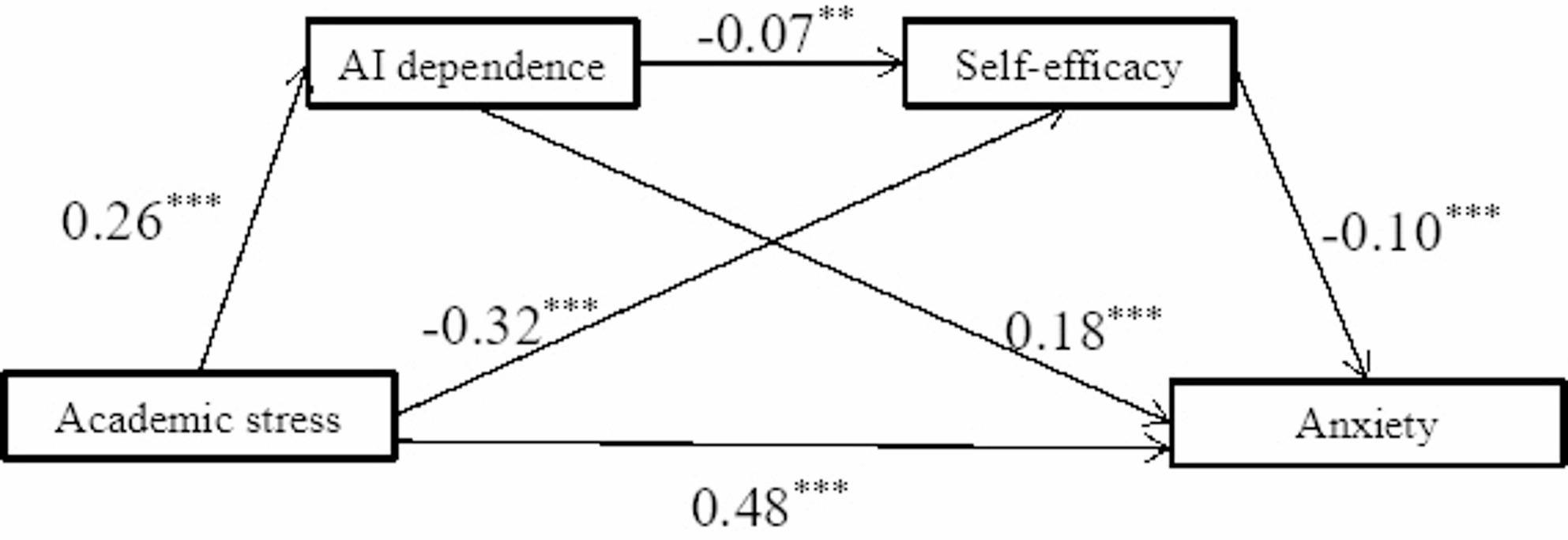
Paths of model 2 (the coefficients are standardized)

Model 2 examined the mediating roles of AI dependence and self-efficacy in the relationship between academic stress and anxiety. The results of the path coefficients are shown in Fig. 2 and Table 4, and the mediation effects are presented in Table 5.

**Table 4:** Results of the chain mediation model 2

Outcome	Predictor	*R*²	F	SE	t	β
Anxiety	Academic stress	0.31	734.61	0.021	27.10	0.558***
AI dependence	Academic stress	0.07	121.26***	0.031	11.01	0.264***
Self-efficacy	Academic stress	0.12	112.49***	0.026	-13.37	-0.323***
	AI dependence			0.020	-3.03	-0.073**
Anxiety	Academic stress	0.35	293.23***	0.023	21.84	0.477***
	AI dependence			0.016	8.43	0.175***
	Self-efficacy			0.020	-4.77	-0.102***

*β *Standardized coefficient

* *p* < 0.05, ** *p* < 0.01, *** *p* < 0.001

**Table 5:** Mediation effect analysis of model 2

Effect Type	Path	Effect	BootSE	LLCI	ULCI
Direct Effect	Academic stress→Anxiety	0.492	0.028	0.438	0.549
Indirect Effect	Academic stress→AI dependence→Anxiety	0.048	0.008	0.033	0.064
	Academic stress→Self-efficacy→Anxiety	0.034	0.008	0.018	0.050
	Academic stress→AI dependence→Self-efficacy→Anxiety	0.002	0.001	0.001	0.004
Total Indirect Effect		0.084	0.012	0.062	0.105
Total Effect		0.575	0.027	0.525	0.628

Bootstrap results based on 5000 samples. CI = 95% Confidence Interval. If 0 is not between LLCI and ULCI, the effect is significant

The same statistical method was repeated, and the results showed that the regression model was significant, with R^2^ = 0.31, F (1, 1621) = 734.61, *p* < 0.001. Academic stress positively predicted AI dependence (*β* = 0.26, *p* < 0.001). AI dependence, in turn, negatively predicted self-efficacy (*β* = -0.07, *p* < 0.01). Finally, self-efficacy negatively predicted anxiety (*β* = -0.10, *p* < 0.001).

#### Direct effect

The direct effect of academic stress on anxiety was 0.49 (95% CI = [0.44, 0.55]), which remained significant after controlling for the mediators.

#### Indirect effects

The total indirect effect through AI dependence and self-efficacy was 0.084 (95% CI = [0.06, 0.11]). It consists of three significant specific indirect pathways: (a) the indirect effect via AI dependence alone was 0.05 (95% CI = [0.03, 0.06]); (b) The indirect effect via self-efficacy alone was 0.03 (95% CI = [0.02, 0.05]); (c) the sequential indirect effect (academic stress→AI dependence→self-efficacy→anxiety) was 0.002 (95% CI = [0.001, 0.004]).

## Discussion

### Direct effect of academic stress on academic burnout and anxiety

The current results indicate that academic stress has a significant and direct influence on academic burnout and anxiety as two negative psychological outcomes among college students. Despite the introduction of the mediating effect of AI dependence and self-efficacy, academic stress was still found to be a strong predictor of academic burnout and anxiety, which implies that academic stress is a fundamental psychological risk factor for the mental well-being and academic performance of students.

Academic stress is a chronic demand that challenges the adaptive capacity of students, making it harder to maintain motivation, focus, and emotional control. Especially the COVID-19 pandemic has significantly intensified academic stress among university students due to increased academic workload, disruptions in learning environments, and prolonged social isolation [6]. Although stress levels have shown some recovery after the pandemic, changes in students’coping patterns and learning behaviors appear to persist, indicating long-term effects on academic adjustment [8].

The strong positive correlation between academic stress and burnout reported in this paper is in line with the findings of previous research. Longitudinal and cross-sectional studies across different cultural contexts have repeatedly shown that academic stress is one of the most powerful predictors of students academic burnout [19, 33]. Furthermore, the direct effect of academic stress on anxiety is consistent with cognitive-behavioral models, which suggest that the prolonged presence of stressors (exams, grades, academic competition, and so on) causes worry, physiological arousal, and negative threat assessment, which in turn leads to increased anxiety level [29].

Notably, the direct effects in the current research indicate that academic stress has an immediate impact on the psychological functioning of students. Academic stress remains an independent predictor of negative psychological outcomes, even in an era where technological aids, such as AI tools, are more accessible to facilitate study. This observation supports the idea that the psychological burden of high academic demands cannot be reduced solely through the help of technology. Rather, structural and pedagogical factors, including curriculum design, assessment practices, and institutional expectations, are still considered to be the major determinants of student well-being.

Furthermore, the fact that both direct and indirect effects exist implies a partial rather than full mediation. It shows that although dependence on AI and self-efficacy contribute to the explanation of the relationship between academic stress and burnout/anxiety, they are insufficient to fully explain this relationship. Other processes, including maladaptive coping skills, perfectionism, and low levels of social support, can also be significant factors. Future studies can embrace integrative models that take into consideration both technological and psycho-social mediators in attempting to explain the stress-mental health relationship in modern academic environments.

### Mediation effect of AI dependence

The current results clearly indicate that AI dependence is a mediating factor between academic stress and both academic burnout and anxiety, highlighting AI dependence as a psychologically significant pathway through which academic stress can evolve into maladaptive academic and emotional health outcomes. Specifically, increased academic stress positively predicted increased dependence on AI tools, which, in turn, may cause higher levels of academic burnout and anxiety, even after controlling for the impact of self-efficacy.

In a stress and coping approach, highly stressed students might consider AI tools as a compensatory coping mechanism to deal with academic workload, time pressure, and performance demands. According to the transactional model of stress and coping, individuals under stress actively seek resources to reduce perceived demands or emotional strain [16]. AI tools, such as large language models, can be used as external cognitive aids to complete tasks and alleviate stress in the short term. However, the current evidence suggests that as this dependence becomes habitual or excessive, it can turn into AI dependence, which may come at a psychological cost rather than a benefit.

The present findings can also be interpreted within the broader framework of cognitive offloading. Prior research has shown that cognitive offloading mediates the relationship between AI tool use and key academic outcomes, such as critical thinking, with greater reliance on AI associated with reduced active cognitive processing [44]. From this perspective, AI dependence may reflect a cognitively mediated coping pattern in which students reduce their cognitive effort by relying on external systems, thereby limiting opportunities for self-regulation and mastery.

In addition, the association between academic stress and anxiety was also mediated by AI dependence. This observation is consistent the recent evidence that excessive dependence on AI could increase uncertainty, performance issues, and evaluative anxiety, especially when students lose confidence in their capabilities in the absence of technology [37]. When students are under high academic stress, AI dependence can lead to a reduced sense of competence due to the fear of failure, or an inability to study independently, which can then give rise to stress-related symptoms.

The mediation effect of AI dependence shows that AI dependence is not only a passive phenomenon that is influenced by academic stress, but also an important contributor to negative academic and psychological consequences. It implies that AI dependence cannot be defined only as a neutral or adaptive learning strategy, but it can function as a maladaptive coping behavior in the case of chronic academic stress. This explanation aligns with the research regarding the negative or excessive use of technology, which suggests that such over-reliance may become dysfunctional when it substitutes, rather than facilitates, self-regulated learning and cognitive processes [23].

These results reveal AI dependence as an important psychological process between academic stress and burnout/anxiety during the digital learning age. Although AI tools have significant potential to facilitate the learning process, excessive use of the tool can also promote stress. This reminds us of the need to differentiate between adaptive AI-assisted learning and maladaptive AI dependence in research and education practice. Interventions to decrease academic burnout and anxiety may thus not just deal with academic stressors, but also encourage reflective, balancing, and autonomy-favoring applications of AI technologies.

### Mediation effect of self-efficacy

The findings of the study have shown that self-efficacy plays an important mediating role in the relationship between academic stress and academic burnout, as well as anxiety. In particular, an increase in academic stress was linked to a decrease in self-efficacy, which further predicted an increase in academic burnout and anxiety. These results demonstrate that self-efficacy is a key psychological process through which academic stress compromises the academic involvement and mental health of students.

Within the social cognitive perspective, self-efficacy indicates individuals’ perceptions of their ability to organize the required actions to cope with potential conditions in the future [2]. Self-efficacy in an academic setting affects the interpretation of stressors, the effort that a student applies, and also their ability to solve a problem. The current findings suggest that academic stress negatively affects student confidence in their academic skills, thus making them susceptible to burnout and anxiety. This pattern is consistent with previous research demonstrating that self-efficacy functions as a protective resource that buffers against stress-related maladaptation [35].

Equally, the mediating effect of self-efficacy was also of high significance in the relationship between academic stress and anxiety. The anxiety cognitive models also focus on the role of low perceived coping ability, which magnifies the threat appraisal and worrying situations during stress [4]. Students with low academic self-efficacy have a greater chance of catastrophizing academic difficulties, perceiving themselves as unable to meet expectations, and predicting failure; all of which further fuels anxiety level. It has been consistently demonstrated that students who have a better sense of self-efficacy express less anxiety about tests and overall anxiety about academic performance, even when they are under great pressure [14, 28].

Besides, self-efficacy continues to play a mediating role when AI dependence was introduced into the model, which revealed its independent effect. This indicates that, regardless of the extent to which students are dependent on external assistance, their personal confidence in their own abilities is also an important factor in psychological success. Academic stress may lead students to use external help, but it is self-efficacy that directly increases burnout and anxiety. This observation is particularly applicable in the modern digital learning setting, where an overabundance of external resources may be accompanied by a decrease in confidence in their personal abilities.

These results reinforce the importance of self-efficacy as a basic psychological resource in higher education. They imply that interventions for academic burnout and anxiety can improve the self-efficacy of students through mastery experiences, formative feedback, autonomy-supportive teaching, and skills training. Improving self-efficacy can potentially shield students against stress-inducing activities, and consequently make them less prone to maladaptive coping strategies, which include over-reliance on technological supports.

### Chain mediation effect of AI dependence and self-efficacy

In addition to the independent mediating effect, the current research demonstrated a significant chain mediating effect that academic stress triggers AI dependence, which in turn negatively affects self-efficacy, followed by academic burnout and anxiety. This points to a progressive psychological mechanism by which academic stress is converted into maladaptive academic and emotional results.

This chain mediation suggests that AI dependence can be interpreted as an upstream process that indirectly influences the mental health of students by undermining their self-efficacy. With high academic stress, students may increasingly tend to use AI tools to cope with academic work, including doing coursework, reading complex material, or managing time constraints. Although this dependence can help to decrease perceived workload in the beginning, in the long run or with excessive dependency, active cognitive involvement and independent problem-solving may be reduced. Consequently, students may question their own academic abilities, and their self-efficacy will decrease. This lack of perceived competence then increases vulnerability to burnout and anxiety.

Cognitive offloading research suggests that frequent reliance on external aids can reduce internal cognitive effort and control of task performance [30]. When students continuously relate positive results to AI support instead of their personal capabilities, mastery experiences, (which are one of the strongest drivers of self-efficacy) will diminish [2]. Therefore, the dependence on AI can indirectly affect the loop, which involves competence, confidence, and effort, by disrupting the feedback system. The current findings demonstrate this mechanism using empirical evidence about a sequential pathway from AI dependence to reduced self-efficacy.

The present findings can also be interpreted within a broader framework in which academic outcomes are shaped through indirect, anxiety-mediated processes. Prior research in higher education has shown that academic stress often influences learning outcomes through intermediate mechanisms related to anxiety and cognitive interference [25]. Consistent with this perspective, the current results suggest that AI dependence may function as part of a cognitively mediated coping process, in which students shift cognitive effort to external systems, thereby reducing active engagement and perceived competence. In turn, diminished self-efficacy may exacerbate vulnerability to academic burnout and anxiety. This interpretation highlights the role of cognitive and emotional processes in translating academic stress into negative outcomes.

From an applied perspective, the chain mediation model has significant implications for educational practice and intervention. It implies that measures against academic burnout and anxiety cannot be addressed solely by restricting AI use or by reducing academic stress separately. Instead, the focus of interventions must be on encouraging reflective and self-efficacy-supportive AI use, encouraging students to implement AI as a scaffold, rather than a substitute for their own cognition and intellectual endeavors. For example, pedagogical designs that require students to critically analyze AI outputs, justify their responses, or solve problems independently, could help maintain the experiences of mastery and protect self-efficacy.

### Strengths and limitations

This study has several notable strengths. First, it adopts a large sample size (*N* = 1,623), which enhances the statistical power and robustness of the findings. Second, the study integrates multiple theoretical perspectives, including Cognitive Offloading Theory and the transactional model of stress and coping, to construct a comprehensive explanatory framework. Third, it extends existing research by conceptualizing AI dependence as a maladaptive coping mechanism and empirically testing a sequential mediation model linking academic stress to academic burnout an anxiety.

Despite its contributions, this study has several limitations: First, the cross-sectional design limits the ability to draw causal inferences regarding the relationships among academic stress, AI dependence, self-efficacy, and psychological outcomes. For instance, lower initial self-efficacy might also drive greater AI dependence as a compensatory strategy; Second, all data were collected through self-report measures, which may introduce common method bias and social desirability effects; Third, the sample was limited to university students in China, which may restrict the generalizability of the findings to other cultural or educational contexts.

### Future research directions

Further research can be conducted using longitudinal designs to trace how AI dependence may reshape students’ self-beliefs and mental health in the long term. It would also be valuable to examine more diverse samples across different cultural and educational settings to enhance generalizability. In addition, integrative models that incorporate mediators based on technology and psychosocial factors can also be employed to provide a more comprehensive explanation of the relationship between academic stress and psychological outcomes. Finally, pedagogical interventions, such as requiring students to critically evaluate AI-generated outputs, can be tested in empirical research to determine whether these methods can help maintain mastery experiences and promote adaptive and autonomy-supportive AI use.

## Conclusion

The academic stress is a major risk factor for academic burnout and anxiety among college students in the digital era. Although technological tools, such as generative AI, have been developed to facilitate learning, students may be involved in a maladaptive loop when over-relying on them as a coping mechanism in the context of high level of academic stress. The current results provide evidence for the presence of a serial mediation pathway: academic stress correlates with AI dependence, which, in turn, decreases the self-efficacy of students, eventually contributing to increased burnout and anxiety. This implies that over-dependence on AI may undermine the mastery experiences that should be acquired to develop academic confidence, resulting in students having fewer internal resources to manage academic challenges. In educational contexts, it is appropriate to promote reflective, autonomy-supportive AI use, positioning AI as a scaffold rather than a substitute.

## Data Availability

The data that support the findings of this study are available on request from the first author.

## References

[1] Akinwale GA, Victor VO, Oloko TO. Developing and validating an Artificial Intelligence Dependence Questionnaire (AIDQ) for undergraduate students. Int J Stud Psychol. 2025;5(3):45–50. https://hdl.handle.net/10520/ejc-ijspsy_v5_n3_a7.

[2] Bandura A. Perceived self-efficacy in the exercise of personal agency. J Appl Sport Psychol. 1990;2(2):128–63. 10.1080/10413209008406426.

[3] Barr N, Pennycook G, Stolz JA, Fugelsang JA. The brain in your pocket: evidence that smartphones are used to supplant thinking. Comput Hum Behav. 2015;48:473–80. 10.1016/j.chb.2015.02.029.

[4] Beck AT, Clark DA. An information processing model of anxiety: automatic and strategic processes. Behav Res Ther. 1997;35(1):49–58. 10.1016/S0005-7967(96)00069-1. 9009043 10.1016/s0005-7967(96)00069-1

[5] Beiter R, Nash R, McCrady M, Rhoades D, Linscomb M, Clarahan M, Sammut S. The prevalence and correlates of depression, anxiety, and stress in a sample of college students. J Affect Disord. 2015;173:90–6. 10.1016/j.jad.2014.10.054. 25462401 10.1016/j.jad.2014.10.054

[6] Bohman A, Eger MA, Hjerm M, Mitchell J. COVID-19-induced academic stress and its impact on life satisfaction and optimism. A panel study of Swedish university students between 2020 and 2022. Eur J High Educ. 2024;14(3):429–50. 10.1080/21568235.2023.2209707.

[7] Bottiani JH, Duran CAK, Pas ET, Bradshaw CP. Teacher stress and burnout in urban middle schools: associations with job demands, resources, and effective classroom practices. J Sch Psychol. 2019;77:36–51. 10.1016/j.jsp.2019.10.002. 31837727 10.1016/j.jsp.2019.10.002

[8] Dresen V, Staggl S, Fischer-Jbali L, Canazei M, Weiss E. Stress, burnout and study-related behavior in university students: a cross-sectional cohort analysis before, during, and after the COVID-19 pandemic. Brain Sci. 2025;15(7):718. 10.3390/brainsci15070718. 40722310 10.3390/brainsci15070718 PMC12293200

[9] Dwivedi YK, et al. So what if ChatGPT wrote it? Multidisciplinary perspectives on opportunities, challenges and implications of generative conversational AI for research, practice and policy. Int J Inf Manag. 2023;71:102642. 10.1016/j.ijinfomgt.2023.102642.

[10] Farrokhnia M, Banihashem SK, Noroozi O, Wals A. A SWOT analysis of ChatGPT: Implications for educational practice and research. Innovations Educ Teach Int. 2024;61(3):460–74. 10.1080/14703297.2023.2195846.

[11] Gerlich M. AI tools in society: Impacts on cognitive offloading and the future of critical thinking. Societies. 2025;15(1):6. 10.3390/soc15010006.

[12] Hasin DS, O’Brien CP, Auriacombe M, Borges G, Bucholz K, Budney A, et al. DSM-5 criteria for substance use disorders: recommendations and rationale. Am J Psychiatry. 2013;170:834–51. 10.1176/appi.ajp.2013.12060782. 23903334 10.1176/appi.ajp.2013.12060782 PMC3767415

[13] Hayes AF. Introduction to mediation, moderation, and conditional process analysis: a regression-based approach (3rd ed.). New York: The Guilford Press. http://refhub.elsevier.com/S0001-6918(24)00075-1/rf0130. 2022.

[14] Honicke T, Broadbent J. The influence of academic self-efficacy on academic performance: a systematic review. Educational Res Rev. 2016;17:63–84. 10.1016/j.edurev.2015.11.002.

[15] Kasneci E, et al. ChatGPT for good? On opportunities and challenges of large language models for education. Learn Individual Differences. 2023;103:102274. 10.1016/j.lindif.2023.102274.

[16] Folkman S. "Stress: appraisal and coping." Encyclopedia of behavioral medicine. Cham: Springer International Publishing. 2020:2177–2179. 10.1007/978-3-030-39903-0_215.

[17] Li H, Mei J. Development of stress scale for college student. Chin J Appl Psychol. 2002;8(1):27–32. https://kns.cnki.net/kcms2/article/abstract?v=yqBhao7Q9gzKL1m4_skV4g5lHz8uIGmY2OvDCIJ6JoaAMQ1qjK9QrO4gWDCdXRE5UQAG84MPSjbPMzp5NEFWD-vrrbslMpaY5OHINneqrssH-DTvHBCQ01Lx71t2HNBP3Ftbl3884HThpEboqg2JGNE6pvEyArJEY34E-YYmz7YVnopAEzdmMQ==&uniplatform=NZKPT&language=CHS.

[18] Lian R, Yang L, Wu L. Relationship between professional commitment and learning burnout of undergraduates and scales developing. Acta Physiol Sinica. 2005;37(5):632–6. https://kns.cnki.net/kcms2/article/abstract?v=yqBhao7Q9gwJ8

[19] Lin SH, Huang YC. Life stress and academic burnout. Act Learn High Educ. 2014;15(1):77–90. 10.1177/1469787413514651.

[20] Liu X, Liu Y, Dai Y, et al. Academic stress and university students’ dependency on generative artificial intelligence: a multiple mediation model using PLS-SEM. BMC Psychol. 2026;14:216. 10.1186/s40359-026-03986-9. 41547828 10.1186/s40359-026-03986-9 PMC12895799

[21] Maslach C, Schaufeli WB, Leiter MP. Job burnout. Ann Rev Psychol. 2001;52:397–422. 10.1146/annurev.psych.52.1.397. 11148311 10.1146/annurev.psych.52.1.397

[22] May RW, Bauer KN, Fincham FD. School burnout: Diminished academic and cognitive performance. Learn Individual Differences. 2015;42:126–31. 10.1016/j.lindif.2015.07.015.

[23] Misra S, Cheng L, Genevie J, Yuan M. The iPhone effect: The quality of in-person social interactions in the presence of mobile devices. Environ Behav. 2016;48(2):275–98. 10.1177/0013916514539755.

[24] Morales-García WC, Sairitupa-Sanchez LZ, Morales-García SB, Morales-García M. Development and validation of a scale for dependence on artificial intelligence in university students. Front Educ. 2024;9:1323898. 10.3389/feduc.2024.1323898.

[25] Ouyang H, Xie H, Wang Y, Chen X, Cheng Q, Hou B, Zhang P. Academic stress, cognitive appraisal, academic self-efficacy, and academic anxiety among Chinese college students: a moderated mediation model. Acta Psychol. 2026;263:106322. 10.1016/j.actpsy.2026.106322. 10.1016/j.actpsy.2026.106322 41581350

[26] Owens M, Stevenson J, Hadwin JA, Norgate R. Anxiety and depression in academic performance: an exploration of the mediating factors of worry and working memory. School Psychol Int. 2012;33(4):433–49. 10.1177/0143034311427433.

[27] Pascoe MC, Hetrick SE, Parker AG. The impact of stress on students in secondary school and higher education. Int J Adolescence Youth. 2020;25(1):104–12. 10.1080/02673843.2019.1596823.

[28] Putwain DW, Daly AL. Test anxiety prevalence and gender differences in a sample of English secondary school students. Educational Stud. 2014;40(5):554–70. 10.1080/03055698.2014.953914.

[29] Putwain DW, Wood P, Pekrun R. Achievement emotions and academic achievement: reciprocal relations and the moderating influence of academic buoyancy. J Educ Psychol. 2022;114(1):108. https://psycnet.apa.org/buy/2020-74100-001.

[30] Risko EF, Gilbert SJ. Cognitive offloading. Trends Cogn Sci. 2016;20(9):676–88. 10.1016/j.tics.2016.07.002. 27542527 10.1016/j.tics.2016.07.002

[31] Salmela-Aro K, Read S. Study engagement and burnout profiles among Finnish higher education students. Burnout Res. 2017;7:21–8. 10.1016/j.burn.2017.11.001.

[32] Salmela-Aro K, Upadyaya K. School burnout and engagement in the context of demands–resources model. Br J Educ Psychol. 2014;84(1):137–51. 10.1111/bjep.12018. 24547758 10.1111/bjep.12018

[33] Salmela-Aro K, Upadyaya K, Ronkainen I, Hietajärvi L. Study burnout and engagement during COVID-19 among university students: the role of demands, resources, and psychological needs. J Happiness Stud. 2022;23(6):2685–702. 10.1007/s10902-022-00518-1. 35399578 10.1007/s10902-022-00518-1 PMC8974799

[34] Schaufeli WB, Martínez IM, Pinto AM, Salanova M, Bakker AB. Burnout and engagement in university students. J Cross-Cult Psychol. 2002;33(5):464–81. 10.1177/0022022102033005003.

[35] Schunk DH, DiBenedetto MK. Motivation and social cognitive theory. Contemp Educ Psychol. 2020;60:101832. 10.1016/j.cedpsych.2019.101832.

[36] SchwarzerR, Jerusalem M, editors. Generalized self-efficacy scale. In J. Weinman, S. Wright, & M. Johnston, editors, Measures in health psychology: a user’s portfolio. Causal and control beliefs. Windsor, England: NFER-NELSON. 1995:35–37. https://www.religiousforums.com/data/attachment-files/2014/12/22334_285ebbd019ec89856493442b1b6f9154.pdf.

[37] Shalu, Verma N, Dev K, Bhardwaj AB, Kumar K. The cognitive cost of AI: how AI anxiety and attitudes influence decision fatigue in daily technology use. Annals Neurosciences. 2025;33(1):73–84. 10.1177/09727531251359872. 10.1177/09727531251359872 PMC12367725 40851834

[38] Sparrow B, Liu J, Wegner DM. Google effects on memory: Cognitive consequences of having information at our fingertips. Science. 2011;333(6043):776–8. 10.1126/science.1207745. 21764755 10.1126/science.1207745

[39] Spitzer RL, Kroenke K, Williams JB, Löwe B. A brief measure for assessing generalized anxiety disorder: the GAD-7. Arch Intern Med. 2006;166(10):1092–7. 10.1001/archinte.166.10.1092. 16717171 10.1001/archinte.166.10.1092

[40] Tabachnick BG, Fidell LS, Ullman JB. Using multivariate statistics. Boston, MA: pearson. 2007;5:481–498. https://www.pearsonhighered.com/assets/preface/0/1/3/4/0134790545.pdf.

[41] Tang DD, Wen ZL. Statistical approaches for testing common method bias: problems and suggestions. J Psychol Sci. 2020;1:215223. 10.16719/j.cnki.1671-6981.20200130.

[42] Vasiou A, Vasilaki E, Mastrothanasis K, Gkontelos A. Behind university students’ academic success: exploring the role of emotional intelligence and cognitive test anxiety. Trends High Educ. 2025;4(3):56. 10.3390/higheredu4030056.

[43] Wang H, Tian J. From trust to dependency: how generative AI attributes and organisational support shape student use of generative AI. Educ Inform Technol. 2026;1–24. 10.1007/s10639-026-13983-5.

[44] Xia L, An X, Li X, Dong Y. Perceptions of generative artificial intelligence (AI), behavioral intention, and use experience as predictors of university students’ learning agency in generative AI-supported contexts. J Educational Comput Res. 2026;64(1):92–125. 10.1177/07356331251382853.

[45] Zhang L, Xu J. The paradox of self-efficacy and technological dependence: unraveling generative AI’s impact on university students’ task completion. Internet High Educ. 2025;65:100978. 10.1016/j.iheduc.2024.100978.

[46] Zhang S, Zhao X, Zhou T, et al. Do you have AI dependency? The roles of academic self-efficacy, academic stress, and performance expectations on problematic AI usage behavior. Int J Educational Technol High Educ. 2024;21:34. 10.1186/s41239-024-00467-0.

